# *Lactococcus lactis cremoris* intra-uterine infection: About an uncommon case report

**DOI:** 10.1016/j.ijscr.2022.107077

**Published:** 2022-04-12

**Authors:** Aziz Slaoui, Imane Benmouna, Najia Zeraidi, Amina Lakhdar, Aicha Kharbach, Aziz Baydada

**Affiliations:** a Gynaecology-Obstetrics and Endoscopy Department, Maternity Souissi, University Hospital Center IBN SINA, University Mohammed V, Rabat, Morocco; b Gynaecology-Obstetrics and Endocrinology Department, Maternity Souissi, University Hospital Center IBN SINA, University Mohammed V, Rabat, Morocco

**Keywords:** Intra-uterine infection, *Lactococcus lactis cremoris*, Extraperitoneal cesarean section

## Abstract

**Background:**

When intra-uterine infection (IUI) is suspected or confirmed, intravenous antibiotic therapy providing coverage against common organisms (*S. agalactiae* and *E. coli*) is recommended to be administered immediately in order to reduce the risk of maternal and neonatal infectious complications. Nevertheless, it happens that some infections are due to uncommon microorganisms that do not respond to probabilistic treatment. Therefore, samples with bacteriological examination remain systematic. Moreover, the extraperitoneal cesarean section avoids the opening of the peritoneal cavity used in the Pfannenstiel technique and thus reduces the risk of infectious dissemination.

**Case presentation:**

We hereby present the uncommon case of a 19-year-old primigravida woman who was referred to our facility for acute gastroenteritis at 34 weeks of gestation. The hospital course was complicated by premature rupture of the membranes followed by the development of fever, chills and deterioration of the fetal heart rate (FHR), imposing an urgent extraperitoneal cesarean section for suspected IUI with fetal impact. Bacteriological examination of a placental sample subsequently yielded growth of *Lactococcus lactis cremoris* which makes it to our knowledge the second case reported to date of an IUI due to this bacterium.

**Clinical discussion and conclusion:**

IUI predominantly occurs by ascending bacterial invasion from the lower genital tract to the typically sterile amniotic cavity in the setting of membrane rupture. Extraperitoneal cesarean section serves as a viable alternative to classic transperitoneal delivery in the presence of uterine infection by controlling bacterial spread.

Our case serves as a reminder that IUI can arise from multiple pathogens, including *Lactococcus lactis cremoris* which is known as a harmless bacterium.

## Highlights

• Extraperitoneal caesarean section should be implemented on a widespread basis in intra-uterine infection, avoiding contamination of the peritoneal cavity.
• Extraperitoneal caesarean section offers many advantages in terms of reducing post-operative pain and faster maternal autonomy.
• *Lactococcus lactis cremoris* should no longer be considered as harmless for humans and should at least be suspected in case of a new infection after consumption of unpasteurized dairy products.

## 1 Background

Intra-uterine infection (IUI), also known as chorioamniotitis, is an infection of the fetal membranes and/or the maternal decidua and/or other components of the amniotic cavity, namely the amniotic fluid, placenta, umbilical cord and fetus [1]. In order to reduce the risk of maternal and neonatal infectious complications, intravenous antibiotic therapy providing coverage against common organisms (*S. agalactiae* and *E. coli*) is recommended to be administered immediately whenever an IUI is suspected or confirmed [1], [2]. Once the diagnosis has been established, delivery should be considered. Vaginal delivery being the safer option and cesarean section should be reserved for standard obstetrical indications [1], [2], [3].

We herein present the uncommon case of a 19-year-old primigravida woman who was referred to our facility for acute gastroenteritis at 34 weeks of gestation. The hospital course was complicated by premature rupture of the membranes followed by the development of fever, chills and deterioration of the fetal heart rate (FHR), imposing an urgent extraperitoneal cesarean section for suspected IUI with fetal impact. Bacteriological examination of a placental sample subsequently yielded growth of *Lactococcus lactis cremoris* which makes it to our knowledge the second case reported to date of an IUI due to this bacterium [4].

## 2 Case presentation

We hereby present the case of a 19-year-woman, with no particular pathological history, primigravida primiparous, whose pregnancy was estimated at 34 weeks of gestation and 6 days according to the sonographic assessment within the first trimester, who was referred to our emergency department for apyretic form of acute gastroenteritis. She reported diffuse abdominal pain over the previous 48 h associated with profuse aqueous diarrhea with 6 to 7 stools per day, without vomiting or fever. History of food intake revealed consumption of an unpasteurized buttermilk few hours prior to onset of symptoms.

Upon admission, she was apyretic, normotensive, with no uterine contractions. Per vaginal examination revealed a long, posterior and closed cervix and intact membranes. She initially received standard rapid rehydration using 500 cm^3^ of normal saline as well as symptomatic therapy: 40 mg of Omeprazole per day, 80 mg of Phloroglucinol four times a day and Loperamide 2 mg starting with 2 capsules, then 1 additional capsule after each liquid stool without exceeding 8 capsules a day. A microbiologic stool exam was ordered and a therapeutic trial with Amoxicillin per os was performed, taking into consideration the risk of Listeriosis. Her initial biological assessment was normal. Obstetrical ultrasound showed a monofetal pregnancy with regular cardiac activity, cephalic presenting fetus with an estimated fetal weight of 2630 g at the 61st percentile for gestational age, fundal placenta and normal amniotic fluid volume. Fetal heart rate (FHR) monitoring revealed a sinusoidal pattern with a baseline rate of 145 bpm without decelerations.

**Fig. 1:**
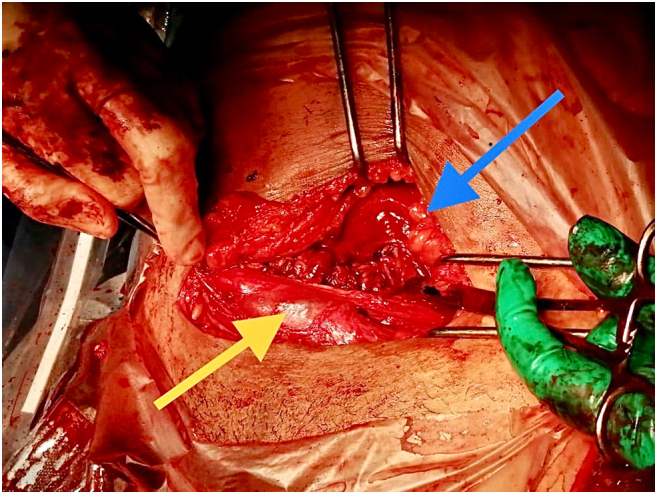
Photography of the extraperitoneal caesarian section. Blue arrow: peritoneal pouch. Yellow arrow: bladder. (For interpretation of the references to colour in this figure legend, the reader is referred to the web version of this article.)

The day after her admission, our patient presented spontaneous premature rupture of the membranes with discharge of a purulent and malodorous amniotic fluid. Physical examination revealed a fever with temperature of 39.4 °C, fundal tenderness and a still closed cervix. FHR monitoring showed severe late decelerations with a nadir of 70 bpm. The diagnosis of intrauterine infection with fetal repercussions drove a decision to proceed with emergency delivery. An extraperitoneal cesarean section by latero-vesical approach was therefore performed (Fig. 1), allowing the birth of a baby girl weighting 2700 g with an Apgar score of 5/7/10 at 1, 5 and 10 min respectively. The newborn was admitted to the neonatal intensive care unit, where she received amoxicillin for 48 h by intravenous route. A biopsy of the placenta was performed and sent to the microbiology laboratory. The cultures obtained revealed a Gram-positive catalase negative coccus. For organism identification, the Api 20 Strep kit (BioMérieux, Marcy l'Etoile, France) was used and *Lactococcus lactis cremoris* was isolated. This bacterium was susceptible to amoxicillin. The patient was therefore managed with simple amoxicillin 1 g 3 times daily for 10 days and became afebrile within 48 h. The postoperative course was uneventful for both mother and child. Thanks to the extraperitoneal technique used for the cesarean section, the patient was able to recover very quickly and started eating the same day. She was discharged from the hospital at D2 postpartum with her newborn.

## 3 Clinical discussion

Initially listed in the genus Streptococcus, it was not until 1985 that *L. lactis* was reclassified in the genus Lactococcus [5]. It is a gram-positive, spherical, homolactate, non-spore forming, facultative anaerobic intestinal bacterium that can be divided into three subspecies: *L. lactis* subsp. lactis, *L. lactis* subsp. cremoris and L. *lactis* subsp. hordniae [6]. The subspecies L. *lactis cremoris* is the most interesting in the cheesemaking industry. They have the advantage of bringing a correct acid production, impeding the growth of undesirable microorganisms thus allowing the preservation, as well as flavor-forming ability as they tend to cause less bitterness [6]. It is commonly considered to be non-pathogenic; however, some human infections have been reported recently regardless of the patient's age, gender and immune status. Therefore, its pathogenic potential is becoming well known by the scientific community. To our knowledge, our case is the second published case of *Lactococcus lactis cremoris* IUI and the 27th worldwide for other infectious sites [4], [7], [8], [9], [10], [11], [12], [13], [14], [15], [16], [17], [18], [19], [20], [21], [22], [23], [24], [25], [26], [27], [28], [29], [30], [31]. Indeed, a review of the literature (Table 1) allowed us to find the 26 cases published before ours and allowed us to highlight some of the features of this uncommon infection.

**Table 1:** Literature review of *Lactococcus lactis cremoris* infection.

Author	Year	Infection site	Sex	Age	Consumption of unpasteurized milk	Dental history	Immune status	Management
Slaoui et al. (our case)	2022	Intra-uterine infection	F	19	Unpasteurized buttermilk	None	Pregnancy	Extraperitoneal cesarean section + antibacterial systemic therapy
Ahmed et al. [7]	2021	Brain abscess	M	18	Unpasteurized milk	None	Normal	Mini-craniotomy for drainage + antibacterial systemic therapy
Fragkiadakis et al. [8]	2017	Liver abscess	M	46	Unpasteurized cheese	Periodontitis	Normal	Percutaneous catheter drainage + antibacterial systemic therapy
Azouzi et al. [4]	2015	Intra-uterine infection	F	32	None	None	Pregnancy	Cesarean section + antibacterial systemic therapy
Buchelli-Ramirez et al. [9]	2013	Necrotising pneumonia	M	70	Yoghurt	None	Normal	Antibacterial systemic therapy
Hadjisymeou et al. [10]	2013	Neck abscess	F	50	Unpasteurized cheese and milk	None	Diabetes mellitus	Incision and drainage + antibacterial systemic therapy
Feierabend et al. [11]	2013	Brain abscess	M	8	None	None	Normal	Drainage by functional endoscopic sinus surgery + antibacterial systemic therapy
Inoue et al. [12]	2012	Subdural empyema	M	33	None	Dental caries	Normal	Open surgery for removal and drainage + antibacterial systemic therapy
Topçu et al. [13]	2011	Brain abscess	M	1	Raw milk products	None	Normal	Craniectomy for drainage + antibacterial systemic therapy
Kim et al. [14]	2010	Liver abscess	M	42	None	None	Normal	Percutaneous catheter drainage + antibacterial systemic therapy
Lin et al. [15]	2010	Endocarditis	M	41	None	None	Normal	Antibacterial systemic therapy
Davies et al. [16]	2009	Ascending cholangitis	F	72	None	None	Normal	Endoscopic sphincterotomy for drainage + antibacterial systemic therapy
Resch et al. [17]	2008	Endocarditis	M	55	Unpasteurized cheese	Dental caries	Normal	Antibacterial systemic therapy
Mofredj et al. [18]	2006	Purulent pleurisy	M	66	Unpasteurized cheese, milk and yoghurt	None	Normal	Percutaneous catheter drainage + antibacterial systemic therapy
Leung et al. [19]	2006	Canaliculitis	F	80	None	Dental caries	Diabetes mellitus	Antibacterial systemic therapy
Zechini et al. [20]	2006	Endocarditis	F	55	None	Dental surgery	Normal	Antibacterial systemic therapy
Koyuncun et al. [21]	2005	Deep neck infection	M	68	Raw milk products	Buccal malignancy mucosa tumor	Previous	Incision and drainage + antibacterial systemic therapy
Antolín et al. [22]	2004	Liver abscess	F	79	None	None	Normal	Percutaneous catheter drainage + antibacterial systemic therapy
Halldórsdóttir et al. [23]	2002	Endocarditis	M	67	Raw milk products	None	Normal	Antibacterial systemic therapy
Akhaddar et al. [24]	2002	Cerebellar abscess	F	45	None	Dental surgery	Normal	Suboccipital craniectomy for drainage + antibacterial systemic therapy
Nakarai et al. [25]	2000	Liver abscess	F	14	None	None	Normal	Percutaneous catheter drainage + antibacterial systemic therapy
Pellizer et al. [26]	1996	Endocarditis	M	56	None	None	Normal	Antibacterial systemic therapy
Durand et al. [27]	1995	Septicemia	M	69	Yoghurt	None	Chronic lymphocytic leukemia	Antibacterial systemic therapy
Campbell et al. [28]	1993	Septic arthritis	F	57	Unpasteurized milk	None	Normal	Antibacterial systemic therapy
Mannion et al. [29]	1990	Endocarditis	F	65	None	None	Normal	Antibacterial systemic therapy
Torre et al. [30]	1990	Necrotizing pneumonitis	M	24	Unpasteurized cheese and milk	None	HIV	Antibacterial systemic therapy
Wood et al. [31]	1955	Endocarditis	M	21	Sour cream	Irritated gum surrounding a non-vital tooth	Normal	Antibacterial systemic therapy

Among these cases, women accounted for 41% of the global total, including our patient, making a sex ratio of 3:2 [4], [10], [16], [19], [20], [22], [24], [25], [28], [29]. The age varies from 1 year, more exactly 19 months [13], to 79 years [22]. Although the number of reported cases is limited for a proper epidemiological analysis, it indicates that the infection can affect men and women almost evenly, from infancy to old age.

Concerning risk factors, some authors have suggested that immunocompromised subjects are more susceptible to the disease; however, we found only 26% of the cases, including ours, with a compromised immune status [4], [10], [19], [21], [27], [30]. Although the evidence for increased susceptibility of pregnant women to infection is quite weak, immunological alterations during this period may impair pathogen clearance [31]. Therefore, we chose to include them in cases of compromised immunity. Pregnancy was reported in 7% of cases whereas history of oro-dental pathologies has been found in 30% of cases [8], [12], [17], [19], [20], [21], [24], [31]. Nonetheless, the most significant risk factor remains the consumption of unpasteurized dairy products, which was found in 52% of patients, including ours [7], [8], [9], [10], [13], [17], [18], [21], [23], [27], [28], [30], [31].

Regarding pathophysiology, the mode of invasion was most often considered to be hematogenous (88%) as in the seven cases reported of endocarditis [15], [17], [20], [23], [26], [29], [31], the five cases of nervous system infection [7], [11], [12], [13], [24], the five cases of hepatobiliary system infection [8], [14], [16], [22], [25], the three cases of pleuropulmonary infection [9], [18], [30], the first case of IUI [4] as well as in the cases of articular infection [28], ocular infection [19] and septicemia [27]. But it could also occur by loco-regional spread as in the two cases of cervical soft tissue abscesses from a potential oral site infection [10], [21]. Our patient presented with gastroenteritis with profuse diarrhea prior to premature rupture of the membranes. This suggests that the occurrence of IUI may result from an ascending infection in the setting of membrane rupture.

All authors reported collecting appropriate microbiological samples prior to the administration of an empiric antimicrobial therapy, which made it possible to obtain cultures confirming the diagnosis of *Lactococcus lactis cremoris* infection. It is interesting to note that all the antibiotic susceptibility tests that were reported in the literature were in line with a bacterial sensitivity to penicillin and other families of antibiotics, namely aminoglycosides and glycopeptides. Although antibiotic regimens based on the result of susceptibility tests are the mainstay of treatment, fifteen cases (56%), including ours, have reported the need for associated surgical management due to the severity of the abscess in relation to its location, to its size or even its compressive effect [4], [7], [8], [10], [11], [12], [13], [14], [16], [18], [21], [22], [24], [25].

The extraperitoneal cesarean section was first described in 1823 by Baudelocque Auguste, and was in fact a vaginotomy carried out by an upper and extraperitoneal route [32], [33], [34]. It was not until 1909 that W. Latzko et al. [34] described a latero-vesical approach. This operative technique was promoted in the middle of the last century as it offers many advantages in terms of avoiding contamination of the peritoneal cavity with infected amniotic fluid, patient's comfort and quick post-operative autonomy, making this ambulatory technique an interesting alternative to the classic transperitoneal approach [35], [36].

In the mid-1990s, Fauck et al. [37] described a new modified extraperitoneal technique that consisted essentially of a paramedian vertical opening of the fascia, a left paravesical extraperitoneal approach of the uterus, and a purse-string closure of the uterine wall, providing good control of bleeding and decrease of the uterine wound length while increasing its thickness. Twenty years later, Ami et al. [38] described an innovative extraperitoneal approach to CS that can be used on an ambulatory mode under the name of the French ambulatory cesarean section (FAUCS). Being associated with less need for intravenous painkillers, shorter hospital stays and earlier returns to home, make this ambulatory technique an interesting alternative to the classic technique [38], [39].

Several authors [38], [39] concluded that FAUCS represents a viable alternative to transperitoneal delivery in the presence of uterine infection, presumed or proven, as in the Azouzi et al. [4] case and ours.

## 4 Conclusions

Intra-uterine infection predominantly occurs by ascending bacterial invasion from the lower genital tract to the typically sterile amniotic cavity in the setting of membrane rupture. Extraperitoneal cesarean section serves as a viable alternative to classic transperitoneal delivery in the presence of uterine infection by controlling bacterial spread.

Our case serves as a reminder that IUI can arise from multiple pathogens, including *Lactococcus lactis cremoris* which is known as a harmless bacterium.

This work has been reported in line with the SCARE 2020 criteria [40].

## Abbreviations

IUI: intra-uterine infection
FHR: fetal heart rate
FAUCS: French AmbUlatory Cesarean Section
CS: Cesarean Section

## Provenance and peer review

Not commissioned, externally peer-reviewed.

## Availability of data and materials

Supporting material is available if further analysis is needed.

## Funding

There are no funding sources to be declared.

## Consent for publication

Written informed consent was obtained from the patient for publication of this case report and any accompanying images. A copy of the written consent is available for review by the Editor-in-Chief of this journal on request.

## Ethics approval and consent to participate

Ethics approval has been obtained to proceed with the current study. Written informed consent was obtained from the patient for participation in this publication.

## Author contribution

Aziz SLAOUI: study concept and design, data collection, data analysis and interpretation,

writing the paper

Imane BENMOUNA: study design, data collection, data interpretation, writing the paper

Najia ZERAIDI: study design, data collection, data interpretation, writing the paper

Amina LAKHDAR: study design, data collection, data interpretation, writing the paper

Aicha KHARBACH: study design, data collection, data interpretation, writing the paper

Aziz BAYDADA: study concept, data collection, data analysis, writing the paper

## Research registration

Not applicable.

## Guarantor

The corresponding author is the guarantor of submission.

## Declaration of competing interest

The authors declare that they have no competing interests.
